# Heterogeneity of *Clostridioides difficile* asymptomatic colonization prevalence: a systematic review and meta-analysis

**DOI:** 10.1186/s13099-024-00674-0

**Published:** 2025-01-27

**Authors:** Daniel De-la-Rosa-Martínez, Rodrigo Villaseñor-Echavarri, Diana Vilar-Compte, Virna Mosqueda-Larrauri, Paola Zinser-Peniche, Seth Blumberg

**Affiliations:** 1 Francis I Proctor Foundation, University of California San Francisco, 490 Illinois St, San Francisco, CA 94158 USA; 2 Department of Infectious Diseases, Instituto Nacional de Cancerología, Mexico City, Mexico; 3 Department of Medicine, University of California San Francisco, San Francisco, CA USA

**Keywords:** *Clostridioides difficile*, Asymptomatic infection, Colonization, Asymptomatic carrier, Prevalence

## Abstract

**Background:**

Asymptomatic carriers significantly influence the transmission dynamics of *C. difficile*. This study aimed to assess the prevalence of toxigenic *C. difficile* asymptomatic colonization (tCDAC) and investigate its heterogeneity across different populations. We searched MEDLINE, Web of Science, and Scopus for articles published between 2000 and 2023 on tCDAC. Studies including asymptomatic adults with laboratory-confirmed tCDAC were eligible. We performed a random-effects meta-analysis to estimate the pooled prevalence by clinical characteristics, settings, and geographic areas. In addition, we used outlier analyses and meta-regression to explore sources of prevalence variability.

**Results:**

Fifty-one studies involving 39,447 patients were included. The tCDAC prevalence ranged from 0.5 to 51.5%. Among pooled estimates, a high prevalence was observed in patients with cystic fibrosis, outbreak settings, and cancer patients, whereas the lowest rates were found in healthy individuals and healthcare workers. Similar colonization rates were observed between admitted and hospitalized patients. Our meta-regression analysis revealed lower rates in healthy individuals and higher rates in cystic fibrosis patients and studies from North America. Additionally, compared with that among healthy individuals, the prevalence significantly increased by 15–47% among different populations and settings.

**Conclusion:**

Our study revealed that tCDAC is a common phenomenon. We found high prevalence estimates that showed significant variability across populations. This heterogeneity could be partially explained by population characteristics and settings, supporting their role in the pathogenesis and burden of this disease. This highlights the need to identify high-risk groups to improve infection control strategies, decrease transmission dynamics, and better understand the natural history of this disease.

**Supplementary Information:**

The online version contains supplementary material available at 10.1186/s13099-024-00674-0.

## Introduction

*Clostridioides difficile* (CD) is an anaerobic, gram-positive, and spore-forming bacterium responsible for a broad clinical spectrum collectively referred to as *Clostridioides difficile* infection (CDI). Symptoms include acute episodes of diarrhea, fever, nausea, abdominal pain, and life-threatening complications such as colon perforation, toxic megacolon, and sepsis [1]. Despite its potential to cause symptomatic disease, *C. difficile* can also be present in the gut microbiota of asymptomatic carriers [2].

Asymptomatic carriers could play a significant role in the transmission dynamics of *C. difficile*. In this context, these individuals have the potential to serve as reservoirs of infection, contributing to disease endemicity and facilitating both community and nosocomial transmission. This is supported by evidence of bacterial shedding, environmental contamination among the colonized population, and genetic linkage between isolates from asymptomatic carriers and those associated with CDI-related diarrhea [3–8]. Additionally, the asymptomatic population poses a potential risk of progressing to symptomatic disease, which would directly exacerbate the burden of CDI in healthcare facilities and other settings [9].

Although estimating the burden of *C. difficile* asymptomatic colonization (CDAC) could be relevant for reducing and improving our understanding of *C. difficile* transmission dynamics, this has not been fully characterized. Current evidence reveals a wide range of colonization prevalence across different populations and settings [10, 11]. These heterogeneous estimates complicate the accurate assessment of the true burden of colonization, but they also present an opportunity to improve infection control strategies and enhance our understanding of the factors associated with colonization, helping to address important research gaps related to *C. difficile* [2].

In this work, we provide insights into *C. difficile* colonization by conducting a systematic review and meta-analysis to summarize and evaluate published data on toxigenic *C. difficile* colonization while also exploring heterogeneity and prevalence modifiers across different populations and settings.

## Methods

### Search strategy and study selection

We conducted a search via MEDLINE, Web of Science, and Scopus for articles published between January 2000 and December 2023. Since no universal definition for CDAC has been accepted, we included the following keywords to refer to this condition: (“*Clostridioides difficile*” OR “*Clostridium difficile*”) AND (asymptomatic OR colonization OR carrier) AND (prevalence). Languages were restricted to English, Spanish, and French. Additionally, manual screening of literature references from review articles was performed to retrieve articles that met the inclusion criteria.

This review was carried out as recommended by the Meta-analyses of Observational Studies in Epidemiology Guidelines [12]. The Preferred Reporting Items for Systematic Reviews and Meta-Analyses checklist was used to report the findings [13]. This study was registered on the PROSPERO platform (ID CRD42021282347). Ethical approval was not required because this study retrieved data from previously published studies.

### Screening process

Four authors independently reviewed the manuscripts in a two-step process. First, titles and abstracts were screened to identify eligible articles. The full text was subsequently evaluated independently by two investigators to identify those that fulfilled the following inclusion criteria: (a) studies included adults (> 18 years), (b) stool polymerase chain reaction (PCR), enzyme-linked immunosorbent assay (EIA), toxigenic culture, or cell cytotoxicity assay were used for diagnosis, (c) they focused on asymptomatic colonization, (d) studies clearly identified the proportion of asymptomatic carriers of toxigenic strains, and (e) observational studies and clinical trials included at least ten subjects.

Toxigenic *C. difficile* asymptomatic carriers (tCDAC) were defined as those patients in whom toxigenic *C. difficile* was identified by stool PCR, enzyme-linked immunosorbent assay (EIA), toxigenic culture, or cell cytotoxicity assay. Given that bacterial toxins A and B are the primary virulence factors of *C. difficile*, non-toxigenic isolates were excluded from our prevalence estimates. Additionally, our analysis focused exclusively on the burden of colonization in asymptomatic carriers with no diarrhea, a population that is often neglected and excluded from infection control interventions [14].

Since we expected high heterogeneity among the populations, we described and classified studies based on their population and setting characteristics. For this purpose, we used clinical characteristics when studies explicitly restricted screening to select patients with certain comorbidities, including patients with cystic fibrosis, cancer, inflammatory bowel disease (IBD), cirrhosis, or those who had undergone kidney transplantation. The healthy population was treated as another category if studies explicitly mentioned it. Additionally, we formed another group that included individuals at occupational risk, such as healthcare workers.

The elderly population without identifiable comorbidities and residents of long-term care facilities (LTCFs) were classified into one group. In the healthcare context, studies were included in the intensive care unit (ICU) category if the surveillance was restricted to this hospital department. Studies that did not include a clearly differentiated population and could not be classified into the previous categories were grouped under the hospital setting category if surveillance was conducted during the hospital stay or under the hospital admission category if surveillance was performed upon hospital admission. Finally, we also differentiated those studies in which the screening was performed in the context of a hospital *C. difficile* outbreak.

Some studies evaluated the prevalence in two well-differentiated groups, and we treated them as two distinct cohorts. Thus, the number of cohorts included in our analysis was greater than the number of studies included. For example, one study might include a healthy group and a group with a specific comorbidity, which would be considered two independent cohorts within one manuscript. Finally, we also documented the year and region of publication to further describe the study characteristics.

### Quality assessment and data collection

Two authors independently evaluated the relevance and quality of the data using the Joanna Briggs Institute Critical Appraisal Tool [15]. A third member of the research group adjudicated disagreements. To evaluate peer review concordance, the kappa coefficient was calculated for each peer review pair. Data from each included manuscript were extracted and summarized in a standardized database, which included the author, publication date, patient characteristics, comorbidities, and tCDAC prevalence.

### Statistical analysis

We performed random-effects meta-analyses using the inverse variance weighting method to calculate the pooled prevalence. Additionally, the Freeman-Tukey double arcsine transformation was employed for the transformation of proportions, and the restricted maximum likelihood estimator was used for τ² estimation [16]. Confidence intervals were estimated with the Clopper–Pearson method. We assessed the presence of heterogeneity among the included studies using the Q statistic, which evaluates the weighted sum of squared differences between the individual study estimates and the pooled estimator. In the context of a random-effects meta-analysis, weights are adjusted to reflect both the within-study variance and the between-study variability (τ²). The I² statistic was subsequently calculated to quantify the proportion of total variability attributable to heterogeneity, where values of 25%, 50%, and 75% were considered low, moderate, and high heterogeneity, respectively [17].

We expected high heterogeneity in the calculated prevalence estimators. However, we were interested in evaluating the causes of variance in colonization prevalence; thus, additional analyses were performed to explore prevalence variability. We grouped the estimators by population characteristics and clinical settings and then conducted a sensitivity analysis by removing studies identified as outliers on the basis of the following criteria: (1) the lower bound of the confidence interval was above the upper bound of the pooled prevalence confidence interval, or (2) the upper bound of the confidence interval was below the lower bound of the pooled prevalence confidence interval [18].

Similarly, we explored the modifiers of prevalence using mixed effects univariate meta-regression models to assess the impact of study characteristics on the overall pooled prevalence from all included studies. Additionally, we conducted a sub-analysis using healthy individuals as the reference group to evaluate differences in prevalence between the groups. A p value < 0.05 was considered to indicate statistical significance. All analyses were performed in RStudio software (version 2024.04.2 + 764) using *meta* (version 7.0–0) and *metafor* (version 4.6-0) packages.

## Results

**Fig. 1:**
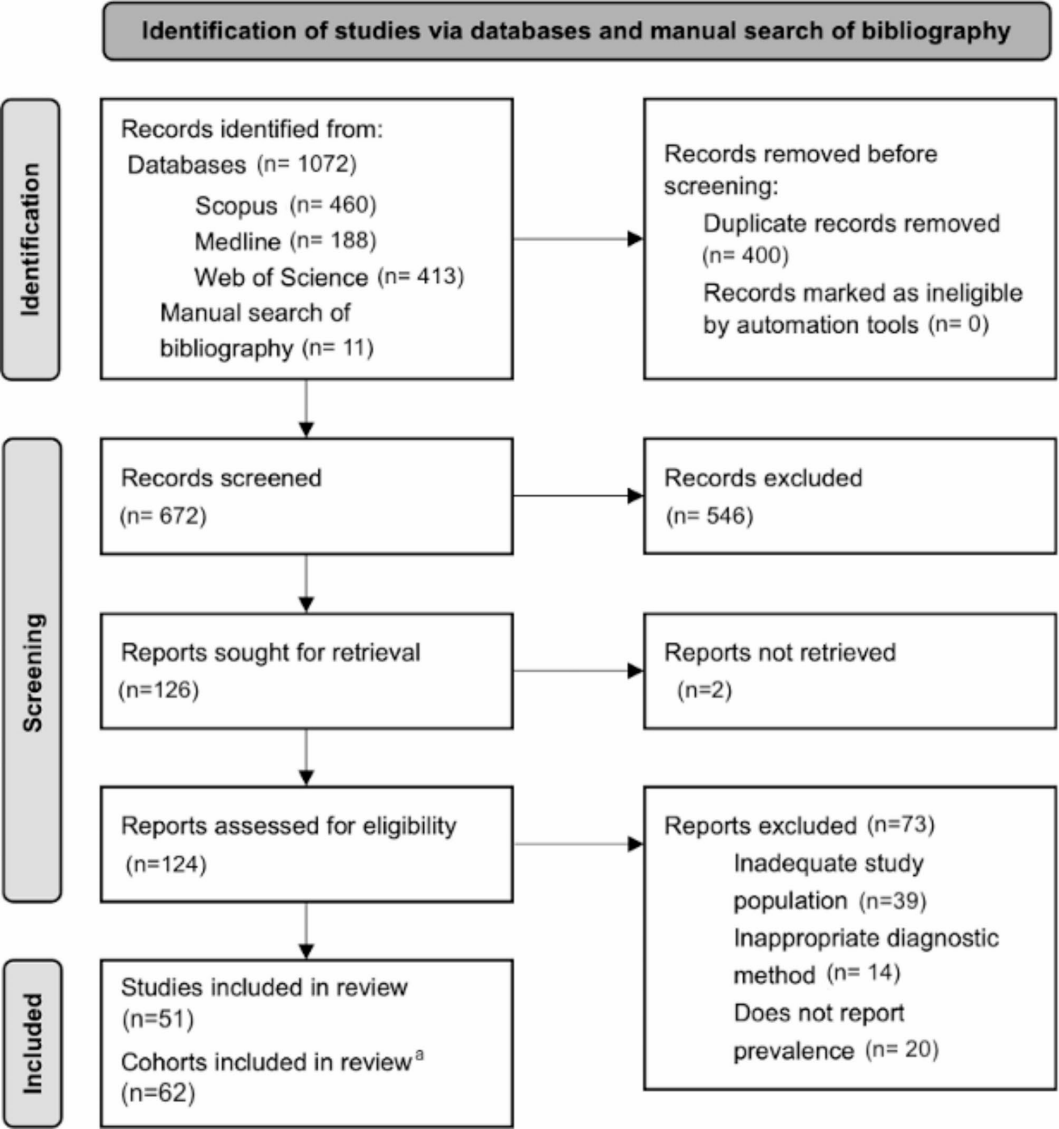
Flowchart for inclusion in this systematic review. ^a^Nine studies reported tCDAC prevalence in two or three well-differentiated populations, resulting in a total of 62 cohorts included in the subsequent analyses

We identified 1072 studies; 946 (88%) were duplicates or nonrelevant at screening; 124 (12%) were reviewed in full text, with 51 (41%) meeting the eligibility criteria for inclusion [3, 5, 8, 19–66]. However, we analyzed 62 cohorts, as 7 studies compared tCDAC prevalence between two well-distinguishable populations, and two included three distinct populations. The identification and selection process are described in Fig. 1. The kappa coefficients were 0.67 and 0.69 for the first and second pairs of reviewers, respectively.

Most of the manuscripts were point-prevalence studies that assessed colonization using a cross-sectional testing approach. Only 12 manuscripts (24%) included more than one test per participant, performed sequentially over time [3, 8, 24, 36, 37, 42, 54–56, 58, 60, 62]. However, the follow-up was inconsistent, ranging from weekly evaluations during hospitalization to repeated testing at discharge. Thus, the amount of transient vs. sustained colonization could not be determined.

In terms of geographic region, 19 (37%) studies were conducted in North America, 18 (35%) in Europe, 10 (20%) in Asia, 3 (6%) in Australia, and 1 (2%) in Africa. According to the publication years, only 2 (4%) manuscripts were published during the decade from 2000 to 2009. In contrast, 37 (72%) studies were published from 2010 to 2019, and 12 (24%) were reported from 2020 to 2023. The year with the highest number of published manuscripts was 2016, with 9 studies, followed by 2017, with 7 studies.

Based on individuals’ characteristics, six (12%) studies included patients with cancer (Study ID 39, 29, 42, 30.2, 35, 33), two (4%) included patients with cystic fibrosis (Study ID 5.2, 49), and one included patients with cirrhosis (2%) (Study ID 48), kidney transplant recipients (2%) (Study ID 46), or patients with IBD (2%) (Study ID 6.2). In the latter group, it is important to note that IBD symptoms could potentially overlap with and complicate the differentiation from CDI cases.

Five (10%) studies included healthy individuals (Study ID 3, 5, 20, 7, 6), including one with healthy pregnant women (Study ID 7.2). Regarding occupational risk, two (4%) studies involved healthcare workers (Study ID 20.2, 28). Details of the individual studies are provided in Table 1.

**Table 1:** Characteristics of identified studies evaluating toxigenic *C. difficile* colonization from 2000 to 2023

Study ID	Autor	Year	Region	Population / Context	*N*	Cases	tCDAC Prevalence
1	Bruijnesteijn et al.	2015	Europe	Individuals seeking general practitioner consultation.	873	4	0.5%
2	Schoevaerdts et al.	2011	Europe	Older adults hospitalized in a geriatric care unit	336	2	0.6%
3	Dong et al.	2018	Asia	Healthy volunteers from different communities in Shanghai	1709	12	0.7%
4	Stuart at al.	2011	Australia	Older adults in three residential care facilities.	119	1	0.8%
5	Burke et al.	2017	Europe	Healthy individuals	99	1	1.0%
6	Clayton et al.	2012	Europe	Healthy individuals.	88	1	1.1%
7	Ye et al.	2016	Asia	Healthy non-pregnant women	651	9	1.4%
7.2	Ye et al.	2016	Asia	Healthy pregnant women.	1009	15	1.5%
8	September et al.	2019	Africa	Residents living in a long-term care facility	119	2	1.7%
9	Meijs et al.	2022	Europe	Veterinary care workers.	482	8	1.7%
10	Skjot-Arkil et al.	2023	Europe	Patients attending emergency departments	5019	89	1.8%
11	Leitner et al.	2020	Europe	Residents of four long-term care facilities	144	3	2.1%
12	Pires et al.	2016	Europe	Patients admitted to acute-care wards at a geriatric hospital.	95	2	2.1%
13	Rabold et al.	2018	Europe	Owners of small companion animals.	578	14	2.4%
14	Kong et al.	2015	North America	Patients screened at admission to six hospitals.	5232	150	2.9%
15	Crobach et al.	2023	Europe	Patients admitted to hospitalization	2211	68	3.1%
16	Jolivet et al.	2022	Europe	Medical, surgery and motherhood hospital wards.	1489	46	3.1%
17	Linsenmeyer et al.	2018	North America	Screening during an outbreak in a healthcare facility.	773	24	3.1%
18	Tschudin-Sutter et al.	2015	North America	Patients admitted to the intensive care unit	542	17	3.1%
16.2	Jolivet et al.	2022	Europe	Long-term care facilities.	390	13	3.3%
19	Meltzer et al.	2019	Asia	All medical patients admitted to the hospital.	2358	81	3.4%
20	Tian et al.	2016	Asia	Healthy adult aged 23–60 years.	1654	60	3.6%
20.2	Tian et al.	2016	Asia	Healthcare workers aged 28–80 years.	348	13	3.7%
21	Zhang et al.	2016	Asia	Adult patients admitted to the intensive care unit.	231	10	4.3%
22	Le Monnier et al.	2022	Europe	Inpatients in medical, surgical, and hematology/transplant wards.	367	16	4.4%
6.2	Clayton et al.	2012	Europe	Outpatients with irritable bowel disease	87	4	4.6%
16.3	Jolivet et al.	2022	Europe	Patients in the intensive care unit.	129	6	4.7%
23	Guerrero et al.	2013	North America	Patients in the intensive care unit.	21	1	4.8%
24	Giufrè et al.	2017	Europe	Older residents of long-term care facilities.	409	20	4.9%
25	Furuya-Kanamori et al.	2017	Australia	Patients admitted to medical, surgical, or intensive care units (median hospital stay of 5 days since admission).	1380	76	5.5%
22.2	Le Monnier et al.	2022	Europe	Inpatients in intensive care wards.	127	9	7.1%
22.3	Le Monnier et al.	2022	Europe	Inpatients in the geriatric/long-term care unit.	227	17	7.5%
26	Worley et al.	2021	North America	Patients in the intensive care unit.	1897	143	7.5%
27	Baron et al.	2020	North America	Patients admitted to an academic medical center.	52	4	7.7%
28	Stojanović et al.	2012	Europe	Medical and paramedical staff at a clinical facility.	63	5	7.9%
29	Cannon et al.	2017	North America	Patients admitted to the hospital’s bone marrow transplant unit for care.	322	30	9.3%
30	Muñoz-Price et al.	2020	North America	Patients admitted to a teaching-affiliated hospital.	2065	194	9.4%
31	Hung et al.	2012	Asia	Adults admitted to the medical wards of a regional hospital.	168	16	9.5%
32	Ryan et al.	2010	Europe	Older adults at a continuous care institution	100	10	10.0%
27.2	Baron et al.	2020	North America	Patients admitted to an academic medical center from nursing facilities.	168	17	10.1%
33	Bruminhent et al.	2014	North America	Adults admitted for hematopoietic stem cell transplantation	150	16	10.7%
34	Arvand et al.	2018	Europe	Adult patients from five rehabilitation clinics.	305	34	11.2%
35	Jain et al.	2016	North America	Recipients of hematopoietic stem cell transplants at a cancer center.	150	18	12.0%
30.2	Muñoz-Price et al.	2020	North America	Patients admitted to the Hematology-Oncology unit	978	124	12.7%
36	Kundrapu et al.	2016	North America	Inpatients from seven hospital wards.	250	32	12.8%
37	Paquet-Bolduc et al.	2018	North America	All patients present in outbreak wards from two academic hospitals.	114	15	13.2%
38	Hung et al.	2013	Asia	Adults admitted to the medical wards of a district hospital.	441	58	13.2%
23.2	Guerrero et al.	2013	North America	Hospitalized adult patients in the spinal cord injury unit or in medical or surgical wards.	128	17	13.3%
39	Vaughn et al.	2018	North America	Patients admitted for scheduled chemotherapy or stem cell transplantation.	101	14	13.9%
40	Halstead et al.	2019	Europe	Older residents of intermediate care facilities.	151	22	14.6%
41	Alasmari et al.	2014	North America	Newly admitted patients to general medical and surgical services	259	40	15.4%
42	Zheng et al.	2017	Asia	Patients with colorectal cancer in the preoperative stage	205	32	15.6%
43	Dubberke et al.	2015	North America	Adults admitted to general medical or surgical wards in a tertiary care hospital	235	37	15.7%
44	Marciniak et al.	2006	North America	Patients admitted to two inpatient acute rehabilitation units for care.	54	9	16.7%
45	Nissle et al.	2016	Europe	Patients admitted to a geriatric unit.	255	43	16.9%
46	Westblade et al.	2019	North America	Kidney transplant recipients in the first 10 days post-transplant.	142	24	16.9%
47	Mi et al.	2020	Asia	Patients admitted to the intensive care unit.	531	93	17.5%
48	Yan et al.	2017	Asia	Patients hospitalized with a diagnosis of hepatic cirrhosis	526	104	19.8%
49	Tai et al.	2021	Australia	Patients living with cystic fibrosis	46	14	30.4%
5.2	Burke et al.	2017	Europe	Adult patients with cystic fibrosis who were pre-lung transplant.	60	19	31.7%
50	Haran et al.	2021	North America	Older adults living in nursing homes	167	78	46.7%
51	Riggs et al.	2007	North America	Residents of a long-term care facility during an outbreak	68	35	51.5%

Thirteen studies (25%) reported prevalence rates in the geriatric population or LTCFs (Study IDs: 50, 11, 40, 27.2, 24, 45, 12, 32, 8, 4, 2, 22.3, 16.2), and seven studies (14%) focused on the intensive care unit (Study IDs: 47, 26, 21, 23, 18, 22.2, 16.3). Manuscripts that did not represent one identifiable characteristic or comorbidity were classified as either hospitalized individuals if the screening was performed during the hospital stay (Study IDs: 36, 23.2, 25, 34, 22, 16) or as hospital admission if the screening occurred at admission or during the early period of hospitalization (Study IDs: 19, 27, 14, 41, 44, 30, 15, 43, 31, 38). In four (8%) manuscripts, patients could not be classified into the mentioned groups: two involved veterinarians or community individuals with close contact with small companion animals (Study IDs: 13, 9), one included community patients attending to their general practitioners (Study IDs: 1), and the other included patients presenting at the emergency departments of eight institutions (Study IDs: 10). Finally, in three (6%) manuscripts (Study IDs: 37, 17, 51), screening was conducted in the context of an outbreak.

### Prevalence of asymptomatic colonization by toxigenic *C. difficile*

Among the 51 studies involving 39,447 patients, 2,091 *C. difficile* asymptomatic carriers were documented. The prevalence of *C. difficile* colonization varied widely across cohorts, ranging from 0.5 to 51%. Individual study prevalence rates and 95% confidence intervals for the included cohorts are depicted in Supplementary Fig. 1.

Our global meta-analysis estimated an overall prevalence of 7.6% (95% CI: 5.7–9.7%). However, a significant degree of heterogeneity was observed (I² = 96%, Q statistic = 1684, *p* < 0.001). Although this heterogeneity decreased, it remained high even after the sensitivity analysis for outliers (I² = 73%, Q statistic = 99, *p* < 0.001).

To address the heterogeneity in our estimation, we performed a subgroup analysis and conducted 9 separate meta-analyses based on population characteristics and settings. A complete description of the prevalence estimators and sensitivity outlier analyses is provided in Table 2. Forest plots for subgroup meta-analyses are presented in Supplementary Figs. 2–10.

**Table 2:** Toxigenic *clostridioides difficile* asymptomatic carrier pooled prevalence based on study level characteristics

Population	Cohorts	Sample	tCDAC Pooled Prevalence	I^2b^	Sensitivity Analysis		
					Excluded Studies^c^	tCDAC Pooled Prevalence	I^2^
**All studies**	62	39,447	7.6 [5.7; 9.7]	96%	31	8.2 [6.9; 9.6]	73%
**Clinical characteristic**							
Healthy population	6	5,210	1.5 [0.7; 2.6]	87%	1	0.9 [0.5; 1.6]	20%
Cystic fibrosis	2	106	31.1 [22.6; 40.4]	0%	-	-	-
Cancer population	6	1,906	12.1 [10.5; 13.9]	10%	-	-	-
Healthcare workers	2	411	4.9 [0.2; 9.7]	53%	-	-	-
Cirrhosis	1	526	19.8 [16.5; 23.3]	-	-	-	-
Kidney transplant recipients	1	142	16.9 [11.2; 23.6]	-	-	-	-
Irritable bowel syndrome	1	87	4.6 [1.0; 10.2]	-	-	-	-
**Clinical settings**							
LTCF /Elderly population^a^	13	2,680	7.1 [2.8; 13.1]	96%	2	5.9 [3.2; 9.3]	88%
ICU patients	7	3,478	6.6 [3.5; 10.6]	92%	1	5.0 [3.3; 7.1]	73%
Hospital setting	6	3,919	7.6 [4.3; 11.6]	92%	1	8.8 [5.3; 13.0]	88%
Hospital admission	10	13,075	8.6 [5.3; 12.5]	97%	3	12.2 [9.8; 14.9]	70%
Outbreak	3	955	18.5 [0.5; 52.1]	98%	-	-	-

(a) Most studies included patients over 65 years, although the mean age varied; (b) I^2^ quantifies the proportion of variability in effect estimates across studies that is attributable to heterogeneity; (c) Manuscripts were excluded during the sensitivity outlier analysis. Abbreviations: tCDAC: Toxigenic *C. difficile* asymptomatic carrier, LTCF: Long-term care facility, ICU: Intensive care unit

Among the pooled estimations, the populations with the highest tCDAC prevalences were patients with cystic fibrosis (31.1%; 95% CI: 22.6–40.4; I²= 0%), studies conducted during outbreaks (18.5%; 95% CI: 0.5–52.1; I²= 98%), and patients with cancer (12.1%; 95% CI: 10.5–13.9; I²= 10%). In contrast, healthy individuals (1.5%; 95% CI: 0.7–2.6, I²= 87%) and healthcare workers (4.9%; 95% CI: 0.2–9.7; I²= 53%) showed the lowest colonization rates. Additionally, patients with cirrhosis (19.8%; 95% CI: 16.5–23.3) and kidney transplant recipients (16.9%; 95% CI: 11.2–23.6) also exhibited high prevalence rates of colonization. However, these values were derived from individual studies.

With respect to the healthcare setting, we did not observe differences in prevalence rates among patients at admission (8.6%; 95% CI: 5.3–12.5; I²= 97%), in the ICU (6.6%; 95% CI: 3.5–10.6; I²= 92%), or in hospitalized individuals (7.6%; 95% CI: 4.3–11.6; I²= 92%). Although heterogeneity decreased in all estimates after the sensitivity outlier analysis, it remained high for most of them (Table 2).

Thirty-four (67%) manuscripts provided some information on prior antibiotic use in the tested populations. Among those with available data, prior antimicrobial exposure ranged from 13 to 96%, with particularly high levels observed in post-transplant patients [50, 54], patients with cystic fibrosis [33], and patients admitted to intensive care units [25].

Although antibiotic exposure information was available for most studies, its definition varied widely across manuscripts. Authors used timeframes ranging from one to six months to define prior antibiotic exposure. Moreover, some studies reported general antibiotic use without specifying types, while others detailed individual use of specific antibiotic classes. Additionally, some manuscripts did not stratify overall use by age or by the subpopulations evaluated, making it challenging to derive prior exposure for certain cohorts included in our review. Due to the lack of granularity, we were unable to explicitly include antibiotic exposure in our meta-regression model.

### Meta-regression to identify modifiers of tCDAC prevalence estimates

The subanalysis of specific populations, settings, or locations did not completely address study heterogeneity. Since each study had a combination of factors that could contribute to disease prevalence, we assessed whether meta-regression analysis could explain more of the heterogeneity in tCDAD prevalence (Table 3). Among all included populations, we found that the healthy population had a significantly lower colonization prevalence (coefficient: -0.17, 95% CI: -0.29; -0.06; *p* = 0.004), whereas those with cystic fibrosis had higher colonization rates (coefficient: 0.32, 95% CI: 0.11; 0.53; *p* = 0.003). Publications from North America reported significantly higher colonization rates (coefficient: 0.13, 95% CI: 0.06; 0.20; *p* < 0.001), whereas those published in Europe reported lower colonization rates (coefficient: -0.09, 95% CI: -0.16; -0.02; *p* = 0.013). We did not find differences in colonization rates based on publication date (coefficient: -0.005, 95% CI: -0.01; 0.005; *p* = 0.293).

**Table 3:** Meta-regression results relating study characteristics to asymptomatic carrier prevalence among all included studies

Population characteristics or setting (*n*)	Coefficient	95% CI		*p* value
		Inferior	Superior	
Healthy population (*n* = 6)	-0.17	-0.29	-0.06	0.004
Cancer population (*n* = 6)	0.08	-0.04	0.21	0.177
Cystic Fibrosis (*n* = 2)	0.32	0.11	0.53	0.003
Inflammatory bowel disease (*n* = 1)	-0.06	-0.36	0.25	0.712
Cirrhosis (*n* = 1)	0.18	-0.10	0.47	0.214
Kidney transplant (*n* = 1)	0.14	-0.15	0.44	0.335
Hospital setting – hospitalized patients (*n* = 6)	0.0001	-0.12	0.12	0.999
Hospital setting – patients at admission (*n* = 10)	0.02	-0.08	0.12	0.638
Hospital setting – health care workers (*n* = 2)	-0.04	-0.26	0.17	0.704
Intensive Care Unit (*n* = 7)	-0.02	-0.14	0.10	0.760
Long-term care facilities/Elderly population (*n* = 13)	-0.01	-0.10	0.08	0.813
Outbreak (*n* = 3)	0.16	-0.01	0.33	0.068
**Geographic Region**				
North America (*n* = 19)	0.13	0.06	0.20	<0.001
Australia (*n* = 3)	0.01	-0.16	0.19	0.870
Asia (*n* = 10)	-0.03	-0.12	0.06	0.494
Europe (*n* = 18)	-0.09	-0.16	-0.02	0.013
Africa (*n* = 1)	-0.14	-0.44	0.15	0.348
**Publication year**	-0.005	-0.01	0.005	0.293

Univariate meta-regressions were performed to adjust for the effect of study-level covariates on the overall prevalence estimation. Coefficients indicate the change in prevalence associated with each predictor. Positive coefficients denote a higher prevalence in the group, whereas negative coefficients denote a lower prevalence. Significance was assessed via confidence intervals (CIs) and p-values

Abbreviations: LTCF: Long-term care facility; ICU: intensive care unit

Additionally, compared with the healthy group, certain populations or settings had a significantly greater disease burden. Those with the most pronounced differences were patients with cystic fibrosis (47%; 95% CI: 25–68%, *p* < 0.001), outbreak settings (30%; 95% CI: 13–48%, *p* < 0.001), and patients with cancer (23%; 95% CI: 9–37%, *p* = 0.001). Similarly, patients with cirrhosis (33%; 95% CI: 8–59%, *p* = 0.011) and those with kidney transplants (30%; 95% CI: 3–56%, *p* = 0.029) had significantly higher prevalence rates than healthy individuals. However, these prevalences were obtained from individual studies. No differences were found between healthy individuals and healthcare workers (11%; 95% CI: -9–32%, *p* = 0.272) or patients with inflammatory bowel disease (10%; 95% CI: -18–37%, *p* = 0.481). Additional population comparisons are presented in Table 4.

**Table 4:** Meta-regression comparing *Clostridioides difficile* asymptomatic carrier prevalence of specific populations versus healthy group

Population characteristics (*n*)	Coefficient	95% CI		*p* value
		Inferior	Superior	
Healthy population (*n* = 6)	**Reference**			
Cystic Fibrosis (*n* = 2)	0.47	0.25	0.68	<0.001
Outbreak (*n* = 3)	0.30	0.13	0.48	<0.001
Cancer population (*n* = 6)	0.23	0.09	0.37	0.001
Hospital setting – patients at admission (*n* = 10)	0.17	0.05	0.30	0.006
Hospital setting – hospitalized patients (*n* = 6)	0.15	0.02	0.29	0.029
Long-term care facilities/Elderly population (*n* = 13)	0.15	0.03	0.27	0.017
Intensive Care Unit (*n* = 7)	0.14	0.003	0.28	0.046
Hospital setting – health care workers (*n* = 2)	0.11	-0.09	0.32	0.272
Cirrhosis (*n* = 1)	0.33	0.08	0.59	0.011
Kidney transplant (*n* = 1)	0.30	0.03	0.56	0.029
Inflammatory bowel disease (*n* = 1)	0.10	-0.18	0.37	0.481
*Another setting (*n* = 4)	-0.005	-0.16	0.15	0.953

Meta-regression was performed with the healthy population as the reference group. Model R^2^ = 31%; test of moderators: (QM = 37, *p* < 0.001). *Two studies of veterinarians or community individuals with close contact with small companion animals, one with patients attending their general practitioners and one with patients presenting at the emergency departments, are included

## Discussion

In this study, we performed a systematic review and meta-analysis to assess the prevalence of tCDAC and conducted meta-regression analyses to explore possible causes of prevalence heterogeneity among the published literature.

Other studies have been published regarding the prevalence of asymptomatic carriers. For example, Ziakas et al. and Zacharioudakis et al. reported, in their meta-analyses, a tCDAC pooled prevalence of 14.8% (95% CI: 7.6-24.0%) in long-term care facility residents and 8.1% (95% CI: 5.7-11.1%) for patients at hospital admission [10, 11]. Additionally, previous research focused on pediatric populations estimated a prevalence of 41% (95% CI: 32-50%) in children aged 6 to 12 months, which decreased to 12% (95% CI: 7-18%) among children aged 5 to 18 years [67].

Our study extends this work, as it is one of the few that examines tCDAC prevalence across different populations and settings. While our review estimated a prevalence similar to that previously reported for the adult population [10, 11], this estimate should be interpreted cautiously because of the significant heterogeneity observed across studies. This variability was expected, as specific population characteristics could influence susceptibility to colonization. Therefore, one of our primary objectives was to explore these differences through subgroup and meta-regression analyses to identify the sources of heterogeneity.

After our subgroup analysis, we identified variations in prevalence among different groups, particularly high prevalence rates among the oncologic population, patients with cystic fibrosis, the outbreak setting, and LTCF residents. Although other populations, such as cirrhosis patients and patients with kidney transplants, also presented high prevalence rates, these estimations were based on individual studies. As expected, the group with the lowest tCDAC prevalence was the healthy population. In the meta-regression analysis, we determined that patients with cystic fibrosis had prevalences that significantly differed from the overall prevalence estimate. In addition, when comparing subgroups with the healthy population, we found that the prevalence significantly increased by 15 to 47% among specific groups and settings (Table 4).

Some of the differences observed among particular subgroups could be influenced by the pathophysiology of the disease and specific exposures that condition different degrees of vulnerability to high colonization rates [48]. For example, patients with cystic fibrosis experience microbiome disturbances due to the high use of antibiotics, as well as pH and mucus disturbances in the gastrointestinal tract driven by cystic fibrosis transmembrane conductance regulator dysfunction [68, 69]. Patients with cancer are exposed to cytotoxic therapies that may alter the immunological response associated with colonization pathogenesis. In addition, both of these populations have a high prevalence of risk factors previously associated with *C. difficile* acquisition, such as prior hospitalizations (OR: 2.18; 95% CI: 1.86–2.56; *p* < 0.001), gastric acid suppression therapy (OR: 1.42; 95% CI: 1.17–1.73; *p* < 0.001), tube feeding (OR: 2.02; 95% CI: 1.06–3.85; *p* = 0.030), and steroid use (OR: 1.58; 95% CI: 1.14–2.17; *p* = 0.006) [70].

Antibiotic use plays a critical role in the pathophysiology of *C. difficile* and may contribute to high colonization rate [71]. In this review, although granular data regarding antimicrobial use was not widely available and the definitions of prior exposure were not standardized, overall antibiotic exposure was highly prevalent in some populations with high colonization rates, such as patients with cystic fibrosis [33] and post-transplant individuals with solid organ [50] or hematological malignancies [54]. This likely contributes to colonization susceptibility due to microbiome disruption caused by antimicrobial agents [71].

Older patients have a greater prevalence of comorbidities and more exposure to medical treatments, which could potentially increase the risk of tCDAC and CDI [72]. It is common for this population to live in LTCFs, which may confer higher CDAC risk because of close coexistence in communal housing settings [72, 73]. However, in addition to these examples, the synergistic interaction of multiple factors may be the reason for the higher colonization rates than any individual factor [7, 24].

Although HCWs are generally healthy, they are at increased risk of acquiring tCDAC due to occupational exposure. While the prevalence of colonization was greater among HCWs than among the healthy population, this difference was not statistically significant.

Colonization at admission did not differ from that observed in hospitalized patients and those in the ICU, which is relevant for several reasons. For example, asymptomatic carriers admitted to the hospital could play a significant role in transmission dynamics, potentially serving as reservoirs of infection and contributing to the endemic persistence of the pathogen within healthcare settings. These carriers could directly increase the CDI burden if they progress to symptomatic disease [74]. On the other hand, there is a risk of overdiagnosis, as colonized individuals may develop diarrhea from causes unrelated to CDI. In this context, relying solely on the presence of the bacteria to diagnose CDI could lead to unnecessary antibiotic use, which may negatively impact patients and contribute to antimicrobial resistance in healthcare environments [75].

Understanding the differences and conditions that contribute to varying levels of colonization burden could improve infection control interventions. Additionally, prospective follow-up of colonized individuals could provide valuable insights into the natural history of the disease, helping to identify patients at risk of progressing to symptomatic disease who may benefit from prophylactic treatment or decolonization strategies [2, 11, 76]. A more nuanced understanding of the epidemiology of asymptomatic carriers may also help resolve the controversy regarding the ability to distinguish between colonization and symptomatic *C. difficile* infection [2].

Although this work primarily focuses on the prevalence of asymptomatic carriers, a population that potentially facilitates *C. difficile* transmission dynamics within healthcare settings, we acknowledge that in non-healthcare contexts, such as the community, other *C. difficile* sources may also be relevant, including the burden of colonization in non-human reservoirs such as animals, food, and environment [77–79].

Previous studies have emphasized the high prevalence of toxigenic *C. difficile* in livestock, particularly in poultry (0-100%), pigs (0–96%), horses (4–33%), cattle (2–22%), sheep (0–18%), and goats (0–10%), as well as in companion animals such as cats (4–16%) and dogs (0-100%) [79]. Similarly, despite variability, spores have been detected in seafood (49–75%), meat (0–6%), and vegetables (3–5%) [79]. Interestingly, a significant number of ribotypes identified in these sources correspond to those observed in humans [77, 79].

In the environment, a collection of 7,857 samples from 10 countries across the Americas, Europe, and Asia documented a global prevalence of *C. difficile* as high as 25%, with small variation among healthcare (23%), non-healthcare (23%), and outdoor spaces (25%) [78]. The ribotypes identified in these settings were largely similar, highlighting potential uniformity in how *C. difficile* spreads in these environments [78].

Our study has several limitations. Some prevalence estimates included a wide range of diverse populations, which may have introduced bias. Additionally, most studies have relied on cross-sectional samples from single hospitals or locations over relatively short time frames, which may not accurately reflect the natural spatial‒temporal variation in colonization. Moreover, asymptomatic status was assessed at a single point in time, meaning that progression to symptomatic disease was not considered. As a result, it is possible that we did not identify long-term colonized individuals, and some of them may have been in the incubation period of the disease, potentially being identified later as symptomatic cases. However, the lack of follow-up data does not modify the potential role of colonized individuals in transmission dynamics. While this limitation affects estimates of the duration of infectiousness, it does not alter their potential capacity to shed bacteria during the testing period.

## Conclusion

*C. difficile* asymptomatic colonization is a common phenomenon. In this study, we found that the prevalence of asymptomatic colonization by toxigenic *C. difficile* varied substantially among different populations. This heterogeneity could be partially explained by population characteristics and settings, supporting the significant role that individual and environmental characteristics play in the pathogenesis of this disease. Identifying groups with high colonization rates is crucial for several reasons, including a better understanding of *C. difficile* transmission dynamics, the natural history of the disease, and the improved implementation of infection control strategies.

## Electronic supplementary material

Below is the link to the electronic supplementary material.

Supplementary Material 1

## Data Availability

Data availability statement: All data are available from the cited literature.

## References

[1] Czepiel J, Dróżdż M, Pituch H, et al. *Clostridium difficile* infection: review. Eur J Clin Microbiol Infect Dis. 2019;38(7):1211–21. 10.1007/s10096-019-03539-6. 30945014 10.1007/s10096-019-03539-6 PMC6570665

[2] Crobach MJT, Vernon JJ, Loo VG, et al. Understanding *Clostridium difficile* colonization. Clin Microbiol Rev. 2018;31(2):e00021–17. 10.1128/CMR.00021-17. 29540433 10.1128/CMR.00021-17 PMC5967689

[3] Riggs MM, Sethi AK, Zabarsky TF, Eckstein EC, Jump RLP, Donskey CJ. Asymptomatic carriers are a potential source for transmission of epidemic and nonepidemic *Clostridium difficile* strains among long-term care facility residents. Clin Infect Dis. 2007;45(8):992–8. 10.1086/521854. 17879913 10.1086/521854

[4] Donskey CJ, Sunkesula VCK, Stone ND, et al. Transmission of *Clostridium difficile* from asymptomatically colonized or infected long-term care facility residents. Infect Control Hosp Epidemiol. 2018;39(8):909–16. 10.1017/ice.2018.106. 29848392 10.1017/ice.2018.106

[5] Halstead FD, Ravi A, Thomson N, et al. Whole genome sequencing of toxigenic Clostridium difficile in asymptomatic carriers: insights into possible role in transmission. J Hosp Infect. 2019;102(2):125–34. 10.1016/j.jhin.2018.10.012. 30359648 10.1016/j.jhin.2018.10.012

[6] Curry SR, Muto CA, Schlackman JL, et al. Use of Multilocus Variable Number of Tandem Repeats Analysis Genotyping to determine the role of asymptomatic carriers in Clostridium difficile Transmission. Clin Infect Dis. 2013;57(8):1094–102. 10.1093/cid/cit475. 23881150 10.1093/cid/cit475 PMC3783061

[7] Gilboa M, Houri-Levi E, Cohen C, et al. Environmental shedding of toxigenic *Clostridioides difficile* by asymptomatic carriers: a prospective observational study. Clin Microbiol Infect. 2020;26(8):1052–7. 10.1016/j.cmi.2019.12.011. 31904567 10.1016/j.cmi.2019.12.011

[8] Worley J, Delaney ML, Cummins CK, DuBois A, Klompas M, Bry L. Genomic determination of relative risks for *Clostridioides difficile* infection from asymptomatic carriage in Intensive Care Unit patients. Clin Infect Dis. 2021;73(7):e1727–36. 10.1093/cid/ciaa894. 32676661 10.1093/cid/ciaa894 PMC8678446

[9] Gilboa M, Baharav N, Melzer E, Regev-Yochay G, Yahav D. Screening for asymptomatic *Clostridioides difficile* carriage among hospitalized patients: a narrative review. Infect Dis Ther. 2023;12(9):2223–40. 10.1007/s40121-023-00856-4. 37704801 10.1007/s40121-023-00856-4 PMC10581986

[10] Ziakas PD, Zacharioudakis IM, Zervou FN, Grigoras C, Pliakos EE, Mylonakis E. Asymptomatic Carriers of Toxigenic *C. difficile* in Long-Term Care Facilities: A Meta-Analysis of Prevalence and Risk Factors. Deshpande A, ed. *PLoS ONE*. 2015;10(2):e0117195. 10.1371/journal.pone.0117195 10.1371/journal.pone.0117195 PMC4338134 25707002

[11] Zacharioudakis IM, Zervou FN, Pliakos EE, Ziakas PD, Mylonakis E. Colonization with toxinogenic *C. Difficile* upon hospital admission, and risk of infection: a systematic review and meta-analysis. Am J Gastroenterol. 2015;110(3):381–90. 10.1038/ajg.2015.22. quiz 391. 25732416 10.1038/ajg.2015.22

[12] Stroup DF, Berlin JA, Morton SC, et al. Meta-analysis of observational studies in epidemiology: a proposal for reporting. Meta-analysis of Observational studies in Epidemiology (MOOSE) group. JAMA. 2000;283(15):2008–12. 10.1001/jama.283.15.2008. 10789670 10.1001/jama.283.15.2008

[13] Page MJ, McKenzie JE, Bossuyt PM, et al. The PRISMA 2020 statement: an updated guideline for reporting systematic reviews. BMJ. 2021;372:n71. 10.1136/bmj.n71. 33782057 10.1136/bmj.n71 PMC8005924

[14] McDonald LC, Gerding DN, Johnson S, et al. Clinical practice guidelines for *Clostridium difficile* infection in adults and children: 2017 update by the Infectious Diseases Society of America (IDSA) and Society for Healthcare Epidemiology of America (SHEA). Clin Infect Dis. 2018;66(7):e1–48. 10.1093/cid/cix1085. 29462280 10.1093/cid/cix1085 PMC6018983

[15] critical-appraisal-tools - Critical Appraisal Tools | JBI. Accessed August 5. 2022. https://jbi.global/critical-appraisal-tools

[16] Harrer M, Cuijpers P, Furukawa TA, Ebert DD. Doing Meta-analysis with R: a Hands-On Guide. 1st ed. Chapman & Hall/CRC; 2021. https://www.routledge.com/Doing-Meta-Analysis-with-R-A-Hands-On-Guide/Harrer-Cuijpers-Furukawa-Ebert/p/book/9780367610074.

[17] Higgins JPT. Measuring inconsistency in meta-analyses. BMJ. 2003;327(7414):557–60. 10.1136/bmj.327.7414.557. 12958120 10.1136/bmj.327.7414.557 PMC192859

[18] Viechtbauer W, Cheung MWL. Outlier and influence diagnostics for meta-analysis. Res Synth Method. 2010;1(2):112–25. 10.1002/jrsm.11. 10.1002/jrsm.11 26061377

[19] Leitner E, Schreiner E, Neuhold M, et al. Low prevalence of *Clostridium difficile* colonization in patients in long-term care facilities in Graz, Austria: a point-prevalence study. Am J Infect Control. 2020;48(10):1144–7. 10.1016/j.ajic.2019.12.011. 31917013 10.1016/j.ajic.2019.12.011

[20] Tai AS, Putsathit P, Eng L, et al. *Clostridioides difficile* colonization and infection in a cohort of Australian adults with cystic fibrosis. J Hosp Infect. 2021;113:44–51. 10.1016/j.jhin.2021.03.018. 33775742 10.1016/j.jhin.2021.03.018

[21] September J, Geffen L, Manning K, et al. Colonisation with pathogenic drug-resistant bacteria and *Clostridioides difficile* among residents of residential care facilities in Cape Town, South Africa: a cross-sectional prevalence study. Antimicrob Resist Infect Control. 2019;8:180. 10.1186/s13756-019-0643-y. 31827776 10.1186/s13756-019-0643-y PMC6862804

[22] Yan D, Chen Y, Lv T, et al. *Clostridium difficile* colonization and infection in patients with hepatic cirrhosis. J Med Microbiol. 2017;66(10):1483–8. 10.1099/jmm.0.000596. 28945189 10.1099/jmm.0.000596

[23] Vaughn JL, Balada-Llasat JM, Lamprecht M, et al. Detection of toxigenic *Clostridium difficile* colonization in patients admitted to the hospital for chemotherapy or haematopoietic cell transplantation. J Med Microbiol. 2018;67(7):976–81. 10.1099/jmm.0.000774. 29863458 10.1099/jmm.0.000774 PMC6152365

[24] Haran JP, Ward DV, Bhattarai SK, et al. The high prevalence of *Clostridioides difficile* among nursing home elders associates with a dysbiotic microbiome. Gut Microbes. 2021;13(1):1–15. 10.1080/19490976.2021.1897209. 33764826 10.1080/19490976.2021.1897209 PMC8007149

[25] Mi H, Bao R, Xiao Y, et al. Colonization of Toxigenic *Clostridium difficile* among Intensive Care Unit patients: a Multi-centre cross-sectional study. Front Cell Infect Microbiol. 2020;10:12. 10.3389/fcimb.2020.00012. 32083021 10.3389/fcimb.2020.00012 PMC7002469

[26] Meltzer E, Smollan G, Huppert A, et al. Universal screening for *Clostridioides difficile* in a tertiary hospital: risk factors for carriage and clinical disease. Clin Microbiol Infect. 2019;25(9):1127–32. 10.1016/j.cmi.2019.02.002. 30771530 10.1016/j.cmi.2019.02.002

[27] Baron SW, Ostrowsky BE, Ceresnak J, et al. Screening of *Clostridioides difficile* carriers in an urban academic medical center: understanding implications of disease. Infect Control Hosp Epidemiol. 2020;41(2):149–53. 10.1017/ice.2019.309. 31822302 10.1017/ice.2019.309 PMC7702293

[28] Paquet-Bolduc B, Gervais P, Roussy JF, et al. Detection and isolation of *Clostridium difficile* asymptomatic carriers during Clostridium difficile infection outbreaks: an exploratory study. Clin Infect Dis. 2018;67(11):1781–3. 10.1093/cid/ciy425. 29771298 10.1093/cid/ciy425

[29] Dong D, Ni Q, Wang C, et al. Effects of intestinal colonization by *Clostridium difficile* and Staphylococcus aureus on microbiota diversity in healthy individuals in China. BMC Infect Dis. 2018;18(1):207. 10.1186/s12879-018-3111-z. 29724187 10.1186/s12879-018-3111-z PMC5934869

[30] Linsenmeyer K, O’Brien W, Brecher SM, et al. *Clostridium difficile* ScreenincolonizationzduringDurioutbreaktsettingetting. Clin Infect Dis. 2018;67(12):1912–4. 10.1093/cid/ciy455. 29846539 10.1093/cid/ciy455

[31] Giufrè M, Ricchizzi E, Accogli M, et al. Colonization by multidrug-resistant organisms in long-term care facilities in Italy: a point-prevalence study. Clin Microbiol Infect. 2017;23(12):961–7. 10.1016/j.cmi.2017.04.006. 28412380 10.1016/j.cmi.2017.04.006

[32] Cannon CM, Musuuza JS, Barker AK, et al. Risk of *Clostridium difficile* infection in hematology-oncology patients colonized with Toxigenic C. Difficile. Infect Control Hosp Epidemiol. 2017;38(06):718–20. 10.1017/ice.2017.48. 28397624 10.1017/ice.2017.48 PMC5531265

[33] Burke DG, Harrison MJ, Fleming C, et al. *Clostridium difficile* carriage in adult cystic fibrosis (CF); implications for patients with CF and the potential for transmission of nosocomial infection. J Cyst Fibros. 2017;16(2):291–8. 10.1016/j.jcf.2016.09.008. 27908697 10.1016/j.jcf.2016.09.008

[34] Zheng Y, Luo Y, Lv Y, et al. *Clostridium difficile* colonization in preoperative colorectal cancer patients. Oncotarget. 2017;8(7):11877–86. 10.18632/oncotarget.14424. 28060753 10.18632/oncotarget.14424 PMC5355311

[35] Nissle K, Kopf D, Rösler A. Asymptomatic and yet C. difficile-toxin positive? Prevalence and risk factors of carriers of toxigenic *Clostridium difficile* among geriatric in-patients. BMC Geriatr. 2016;16(1):185. 10.1186/s12877-016-0358-3. 27846818 10.1186/s12877-016-0358-3 PMC5111236

[36] Zhang X, Wang X, Yang J, Liu X, Cai L, Zong Z. Colonization of toxigenic *Clostridium difficile* among ICU patients: a prospective study. BMC Infect Dis. 2016;16:397. 10.1186/s12879-016-1729-2. 27506470 10.1186/s12879-016-1729-2 PMC4977703

[37] Pires D, Prendki V, Renzi G, et al. Low frequency of asymptomatic carriage of toxigenic *Clostridium difficile* in an acute care geriatric hospital: prospective cohort study in Switzerland. Antimicrob Resist Infect Control. 2016;5:24. 10.1186/s13756-016-0123-6. 27280019 10.1186/s13756-016-0123-6 PMC4898375

[38] Kundrapu S, Sunkesula VCK, Jury LA, et al. Do piperacillin/tazobactam and other antibiotics with inhibitory activity against Clostridium difficile reduce the risk for acquisition of *C. Difficile* colonization? BMC Infect Dis. 2016;16:159. 10.1186/s12879-016-1514-2. 27091232 10.1186/s12879-016-1514-2 PMC4835867

[39] Tian Ttian, Zhao J hong, Yang J et al. Molecular Characterization of *Clostridium difficile* Isolates from Human Subjects and the Environment. *PLoS One*. 2016;11(3):e0151964. 10.1371/journal.pone.0151964 10.1371/journal.pone.0151964 PMC4807052 27011211

[40] yong Ye G, Li N, Chen YB, et al. *Clostridium difficile* carriage in healthy pregnant women in China. Anaerobe. 2016;37:54–7. 10.1016/j.anaerobe.2015.11.010. 26633756 10.1016/j.anaerobe.2015.11.010

[41] van Bruijnesteijn LES, Dullaert-de Boer M, Ruijs GJHM, et al. Case-control comparison of bacterial and protozoan microorganisms associated with gastroenteritis: application of molecular detection. Clin Microbiol Infect. 2015;21(6):e5929–19. 10.1016/j.cmi.2015.02.007. 10.1016/j.cmi.2015.02.007 25700890

[42] Kong LY, Dendukuri N, Schiller I, et al. Predictors of asymptomatic *Clostridium difficile* colonization on hospital admission. Am J Infect Control. 2015;43(3):248–53. 10.1016/j.ajic.2014.11.024. 25728150 10.1016/j.ajic.2014.11.024

[43] Alasmari F, Seiler SM, Hink T, Burnham CAD, Dubberke ER. Prevalence and risk factors for asymptomatic *Clostridium difficile* carriage. Clin Infect Dis. 2014;59(2):216–22. 10.1093/cid/ciu258. 24755858 10.1093/cid/ciu258 PMC4133563

[44] Guerrero DM, Becker JC, Eckstein EC, et al. Asymptomatic carriage of toxigenic *Clostridium difficile* by hospitalized patients. J Hosp Infect. 2013;85(2):155–8. 10.1016/j.jhin.2013.07.002. 23954113 10.1016/j.jhin.2013.07.002

[45] Clayton EM, Rea MC, Shanahan F, et al. Carriage of *Clostridium difficile* in outpatients with irritable bowel syndrome. J Med Microbiol. 2012;61(Pt 9):1290–4. 10.1099/jmm.0.040568-0. 22580916 10.1099/jmm.0.040568-0

[46] Ryan J, Murphy C, Twomey C, et al. Asymptomatic carriage of *Clostridium difficile* in an Irish continuing care institution for the elderly: prevalence and characteristics. Ir J Med Sci. 2010;179(2):245–50. 10.1007/s11845-009-0361-1. 19495833 10.1007/s11845-009-0361-1

[47] Marciniak C, Chen D, Stein AC, Semik PE. Prevalence of *Clostridium difficile* colonization at admission to rehabilitation. Arch Phys Med Rehabil. 2006;87(8):1086–90. 10.1016/j.apmr.2006.03.020. 16876554 10.1016/j.apmr.2006.03.020

[48] Furuya-Kanamori L, Clements ACA, Foster NF, et al. Asymptomatic *Clostridium difficile* colonization in two Australian tertiary hospitals, 2012–2014: prospective, repeated cross-sectional study. Clin Microbiol Infect. 2017;23(1):48e. 1-48.e7. 10.1016/j.cmi.2016.08.030 27615716

[49] Muñoz-Price LS, Hanson R, Singh S, et al. Association between Environmental Factors and toxigenic *clostridioides difficile* carriage at Hospital Admission. JAMA Netw Open. 2020;3(1):e1919132. 10.1001/jamanetworkopen.2019.19132. 31922563 10.1001/jamanetworkopen.2019.19132 PMC6991319

[50] Westblade LF, Satlin MJ, Albakry S, et al. Gastrointestinal pathogen colonization and the microbiome in asymptomatic kidney transplant recipients. Transpl Infect Dis. 2019;21(6):e13167. 10.1111/tid.13167. 31502737 10.1111/tid.13167 PMC6917898

[51] Crobach MJT, Hornung BVH, Verduin C, et al. Screening for *Clostridioides difficile* colonization at admission to the hospital: a multi-centre study. Clin Microbiol Infect. 2023;29(7):891–6. 10.1016/j.cmi.2023.02.022. 36871826 10.1016/j.cmi.2023.02.022

[52] Rabold D, Espelage W, Abu Sin M, et al. The zoonotic potential of *Clostridium difficile* from small companion animals and their owners. PLoS ONE. 2018;13(2):e0193411. 10.1371/journal.pone.0193411. 29474439 10.1371/journal.pone.0193411 PMC5825086

[53] Arvand M, Ruscher C, Bettge-Weller G, Goltz M, Pfeifer Y. Prevalence and risk factors for colonization by *Clostridium difficile* and extended-spectrum β-lactamase-producing Enterobacteriaceae in rehabilitation clinics in Germany. J Hosp Infect. 2018;98(1):14–20. 10.1016/j.jhin.2017.07.004. 28705583 10.1016/j.jhin.2017.07.004

[54] Jain T, Croswell C, Urday-Cornejo V, et al. Clostridium Difficile colonization in hematopoietic stem cell transplant recipients: a prospective study of the Epidemiology and outcomes Involving Toxigenic and nontoxigenic strains. Biol Blood Marrow Transpl. 2016;22(1):157–63. 10.1016/j.bbmt.2015.07.020. 10.1016/j.bbmt.2015.07.020 26211988

[55] Tschudin-Sutter S, Carroll KC, Tamma PD, et al. Impact of Toxigenic *Clostridium difficile* colonization on the risk of subsequent C. difficile infection in Intensive Care Unit patients. Infect Control Hosp Epidemiol. 2015;36(11):1324–9. 10.1017/ice.2015.177. 26223207 10.1017/ice.2015.177

[56] Dubberke ER, Reske KA, Seiler S, Hink T, Kwon JH, Burnham CAD. Risk factors for Acquisition and loss of *Clostridium difficile* colonization in hospitalized patients. Antimicrob Agents Chemother. 2015;59(8):4533–43. 10.1128/AAC.00642-15. 25987626 10.1128/AAC.00642-15 PMC4505269

[57] Stojanović P, Stojanović N, Kocic B, Stanković-Đorđević D, Babić T, Stojanović K. Asymptomatic carriers of *clostridium difficile* in Serbian population. Open Med. 2012;7(6):769–74. 10.2478/s11536-012-0067-z.

[58] Hung YP, Tsai PJ, Hung KH, et al. Impact of toxigenic *Clostridium difficile* colonization and infection among hospitalized adults at a district hospital in southern Taiwan. PLoS ONE. 2012;7(8):e42415. 10.1371/journal.pone.0042415. 22876321 10.1371/journal.pone.0042415 PMC3411658

[59] Stuart RL, Kotsanas D, Webb B, et al. Prevalence of antimicrobial-resistant organisms in residential aged care facilities. Med J Aust. 2011;195(9):530–3. 10.5694/mja11.10724. 22060088 10.5694/mja11.10724

[60] Schoevaerdts D, Swine C, Verroken A, Huang TD, Glupczynski Y. Asymptomatic colonization by *Clostridium difficile* in older adults admitted to a geriatric unit: a prospective cohort study. J Am Geriatr Soc. 2011;59(11):2179–81. 10.1111/j.1532-5415.2011.03685.x. 22098041 10.1111/j.1532-5415.2011.03685.x

[61] Bruminhent J, Wang ZX, Hu C, et al. *Clostridium difficile* colonization and disease in patients undergoing hematopoietic stem cell transplantation. Biol Blood Marrow Transpl. 2014;20(9):1329–34. 10.1016/j.bbmt.2014.04.026. 10.1016/j.bbmt.2014.04.026 24792871

[62] Hung YP, Lin HJ, Wu TC, et al. Risk factors of fecal toxigenic or non-toxigenic *Clostridium difficile* colonization: impact of toll-like receptor polymorphisms and prior antibiotic exposure. PLoS ONE. 2013;8(7):e69577. 10.1371/journal.pone.0069577. 23936050 10.1371/journal.pone.0069577 PMC3723847

[63] Le Monnier A, Candela T, Mizrahi A, et al. One-day prevalence of asymptomatic carriage of toxigenic and non-toxigenic *Clostridioides difficile* in 10 French hospitals. J Hosp Infect. 2022;129:65–74. 10.1016/j.jhin.2022.05.011. 35640734 10.1016/j.jhin.2022.05.011

[64] Jolivet S, Couturier J, Grohs P, et al. Prevalence and risk factors of toxigenic *Clostridioides difficile* asymptomatic carriage in 11 French hospitals. Front Med. 2023;10:1221363. 10.3389/fmed.2023.1221363. 10.3389/fmed.2023.1221363 PMC10402895 37547619

[65] Skjøt-Arkil H, Rune Nanthan K, Chen M, Rosenvinge FS. Carrier prevalence of *Clostridioides difficile* in emergency departments and the association of prior antibiotic consumption: a combined cross-sectional and nested case–control study. J Antimicrob Chemother. 2023;78(8):2089–96. 10.1093/jac/dkad213. 37409612 10.1093/jac/dkad213

[66] Meijs AP, Gijsbers EF, Hengeveld PD, et al. Faecal carriage of *Clostridioides difficile* is low among veterinary healthcare workers in the Netherlands. Epidemiol Infect. 2022;150:e63. 10.1017/S0950268822000383. 35296372 10.1017/S0950268822000383 PMC8931804

[67] Tougas SR, Lodha N, Vandermeer B, et al. Prevalence of detection of *Clostridioides difficile* among asymptomatic children: a systematic review and Meta-analysis. JAMA Pediatr. 2021;175(10):e212328. 10.1001/jamapediatrics.2021.2328. 34338715 10.1001/jamapediatrics.2021.2328 PMC8329794

[68] Dorsey J, Gonska T. Bacterial overgrowth, dysbiosis, inflammation, and dysmotility in the cystic fibrosis intestine. J Cyst Fibros. 2017;16(Suppl 2):S14–23. 10.1016/j.jcf.2017.07.014. 28986022 10.1016/j.jcf.2017.07.014

[69] Burke DG, Fouhy F, Harrison MJ, et al. The altered gut microbiota in adults with cystic fibrosis. BMC Microbiol. 2017;17(1):58. 10.1186/s12866-017-0968-8. 28279152 10.1186/s12866-017-0968-8 PMC5345154

[70] Anjewierden S, Han Z, Brown AM, Donskey CJ, Deshpande A. Risk factors for *Clostridioides difficile* colonization among hospitalized adults: a meta-analysis and systematic review. Infect Control Hosp Epidemiol. 2021;42(5):565–72. 10.1017/ice.2020.1236. 33118886 10.1017/ice.2020.1236

[71] Furuya-Kanamori L, Marquess J, Yakob L, et al. Asymptomatic *Clostridium difficile* colonization: epidemiology and clinical implications. BMC Infect Dis. 2015;15(1):516. 10.1186/s12879-015-1258-4. 26573915 10.1186/s12879-015-1258-4 PMC4647607

[72] Jump RL. *Clostridium Difficile* Infectiolder adultsAdults. Aging Health. 2013;9(4):403–14. 10.2217/ahe.13.37. 24955106 10.2217/ahe.13.37 PMC4061705

[73] Jump RLP, Donskey CJ. *Clostridium difficile* in the Long-Term Care Facility: Prevention and Management. Curr Geri Rep. 2015;4(1):60–9. 10.1007/s13670-014-0108-3. 10.1007/s13670-014-0108-3 PMC4322371 25685657

[74] McLure A, Clements ACA, Kirk M, Glass K. Healthcare-Associated *Clostridium difficile* infections are sustained by Disease from the community. Bull Math Biol. 2017;79(10):2242–57. 10.1007/s11538-017-0328-8. 28776206 10.1007/s11538-017-0328-8

[75] Polage CR, Gyorke CE, Kennedy MA, et al. Overdiagnosis of *Clostridium difficile* infection in the Molecular Test Era. JAMA Intern Med. 2015;175(11):1792. 10.1001/jamainternmed.2015.4114. 26348734 10.1001/jamainternmed.2015.4114 PMC4948649

[76] Schäffler H, Breitrück A. *Clostridium difficile* – From Colonization to Infection. Front Microbiol. 2018;9:646. 10.3389/fmicb.2018.00646. 29692762 10.3389/fmicb.2018.00646 PMC5902504

[77] Lim SC, Knight DR, Riley TV. *Clostridium difficile* and One Health. Clin Microbiol Infect. 2020;26(7):857–63. 10.1016/j.cmi.2019.10.023. 31682985 10.1016/j.cmi.2019.10.023

[78] Jo J, Gonzales-Luna AJ, Lancaster CK, et al. Multi-country surveillance of *Clostridioides difficile* demonstrates high prevalence of spores in non-healthcare environmental settings. Anaerobe. 2022;75:102543. 10.1016/j.anaerobe.2022.102543. 35227896 10.1016/j.anaerobe.2022.102543 PMC9197859

[79] Mastrantonio P, Rupnik M, editors. Updates on Clostridium Difficile in Europe: Advances in Microbiology, Infectious Diseases and Public Health Volume 8. Volume 1050. Springer International Publishing; 2018. 10.1007/978-3-319-72799-8.

