# Supplementary material for: Heterogeneity of Clostridioides difficile asymptomatic colonization prevalence: a systematic review and meta-analysis
Source: Gut Pathog. 2025 Jan 27;17:6. doi: 10.1186/s13099-024-00674-0 (PMC11773978; doi:10.1186/s13099-024-00674-0)

**Supplementary Material**

**Supplementary Figure 1. Prevalence of toxigenic Clostridioides difficile colonization among the included studies**

**
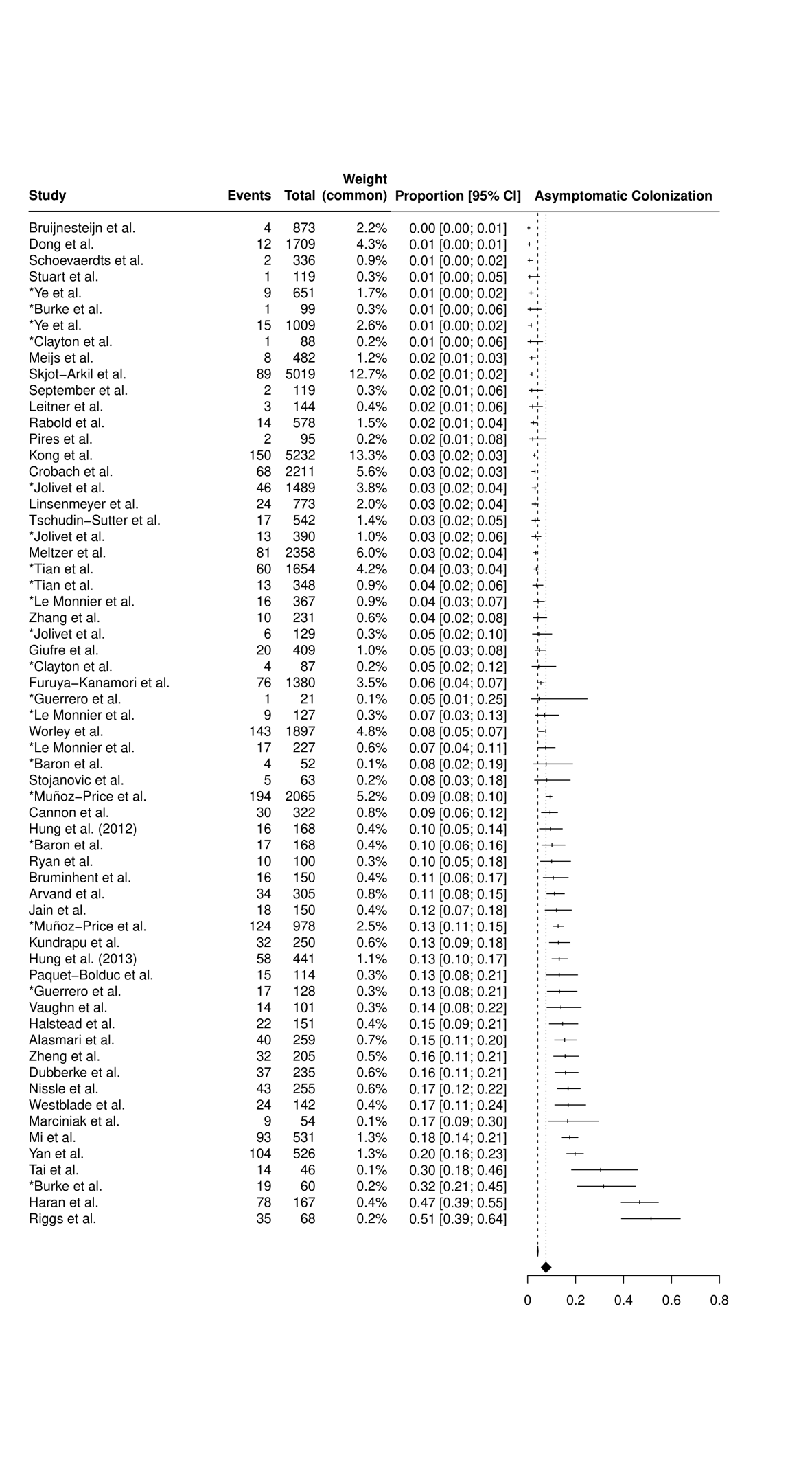
**

*Articles that assessed prevalence in two well-differentiated groups were treated as two separate cohorts. Consequently, while 51 manuscripts were included, the analysis comprises 62 distinct cohorts of individuals.

**Supplementary figure 2. Asymptomatic *C. difficile* colonization among healthy population**


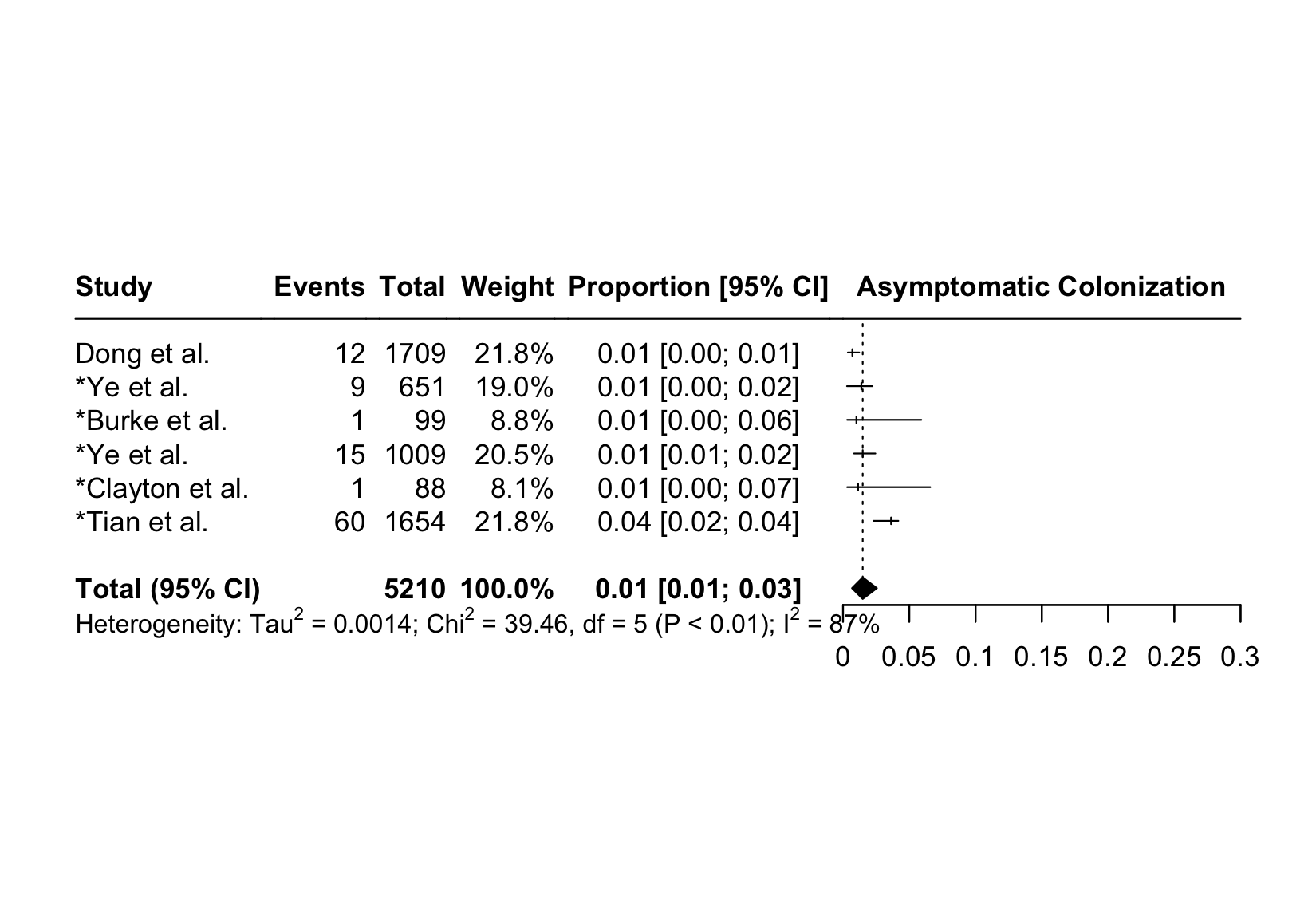


**Supplementary figure 3. Asymptomatic *C. difficile* colonization among long-term care facilities and elderly population.**


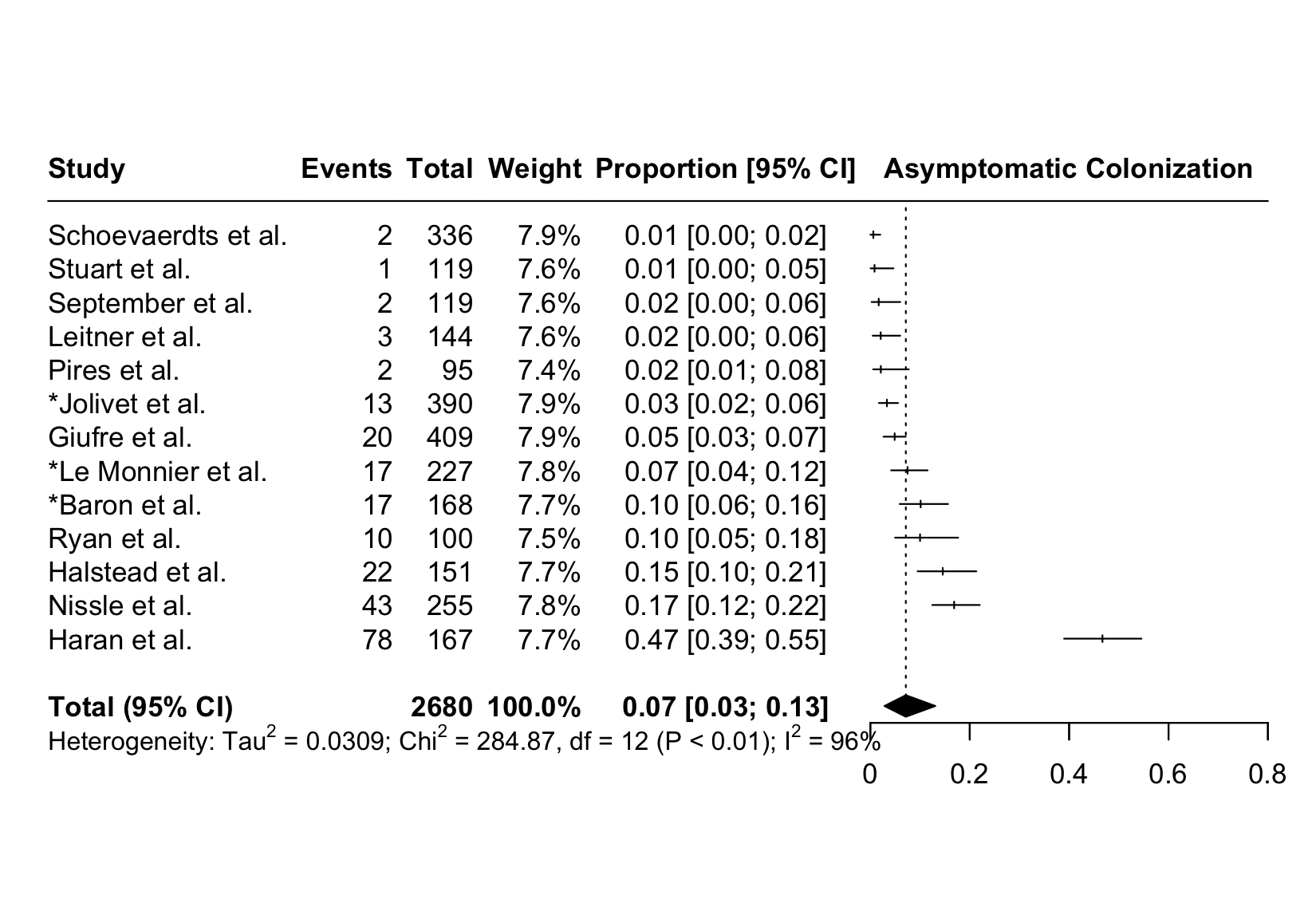


**Supplementary figure 4. Asymptomatic *C. difficile* colonization among patients with cancer**


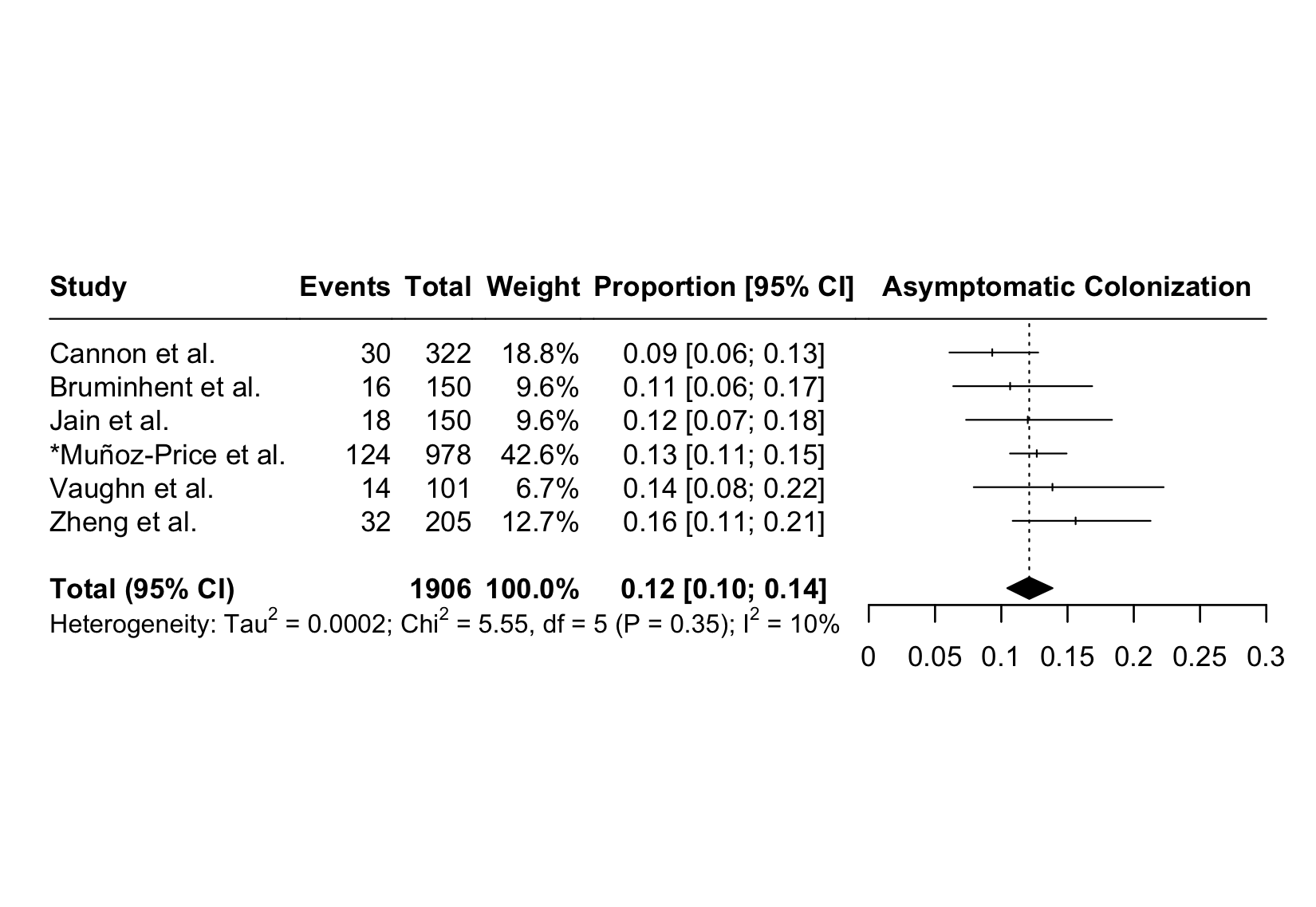


**Supplementary figure 5. Asymptomatic *C. difficile* colonization among patients in the intensive care unit.**


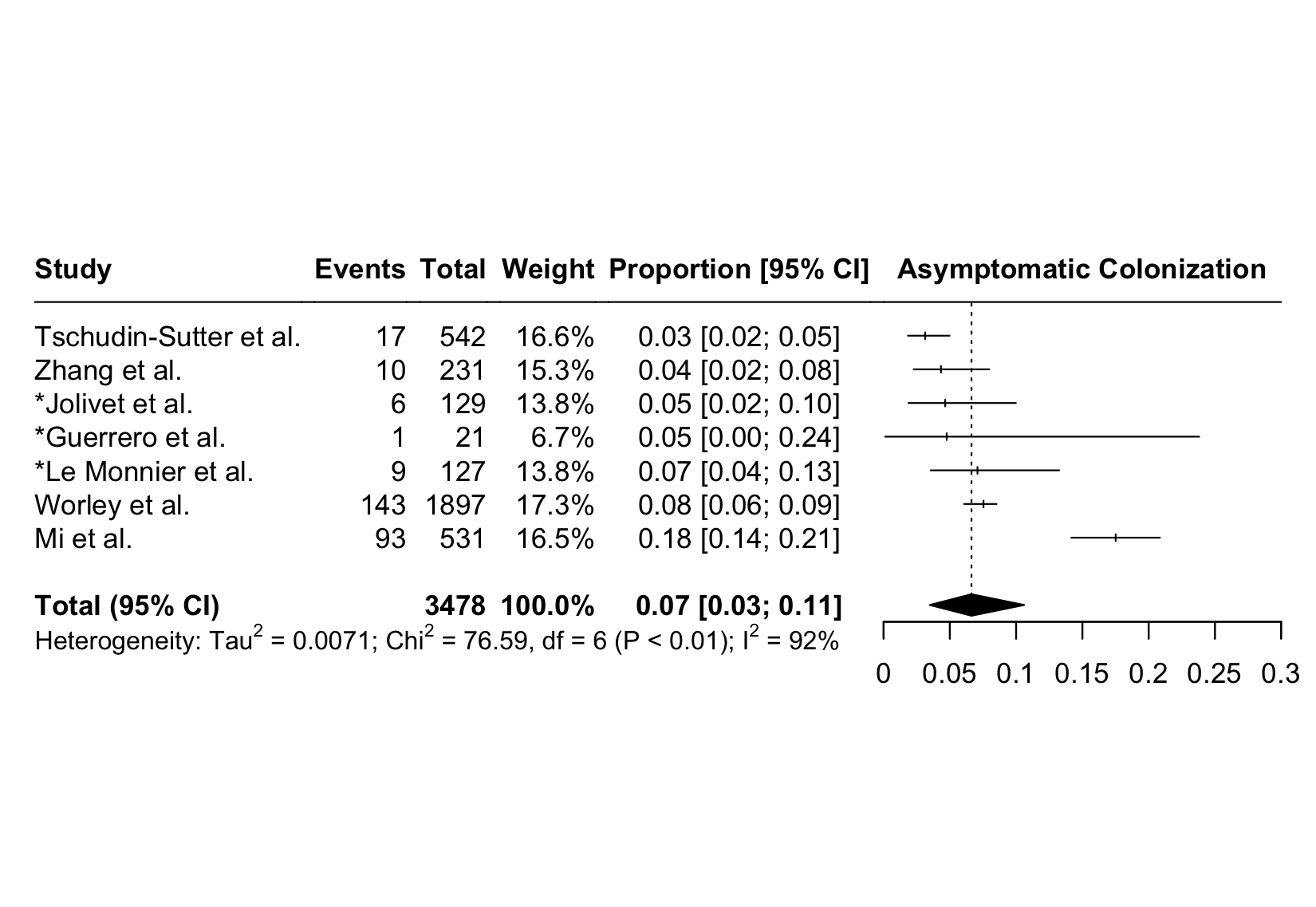


**Supplementary figure 6. Asymptomatic *C. difficile* colonization among patients in hospital setting**


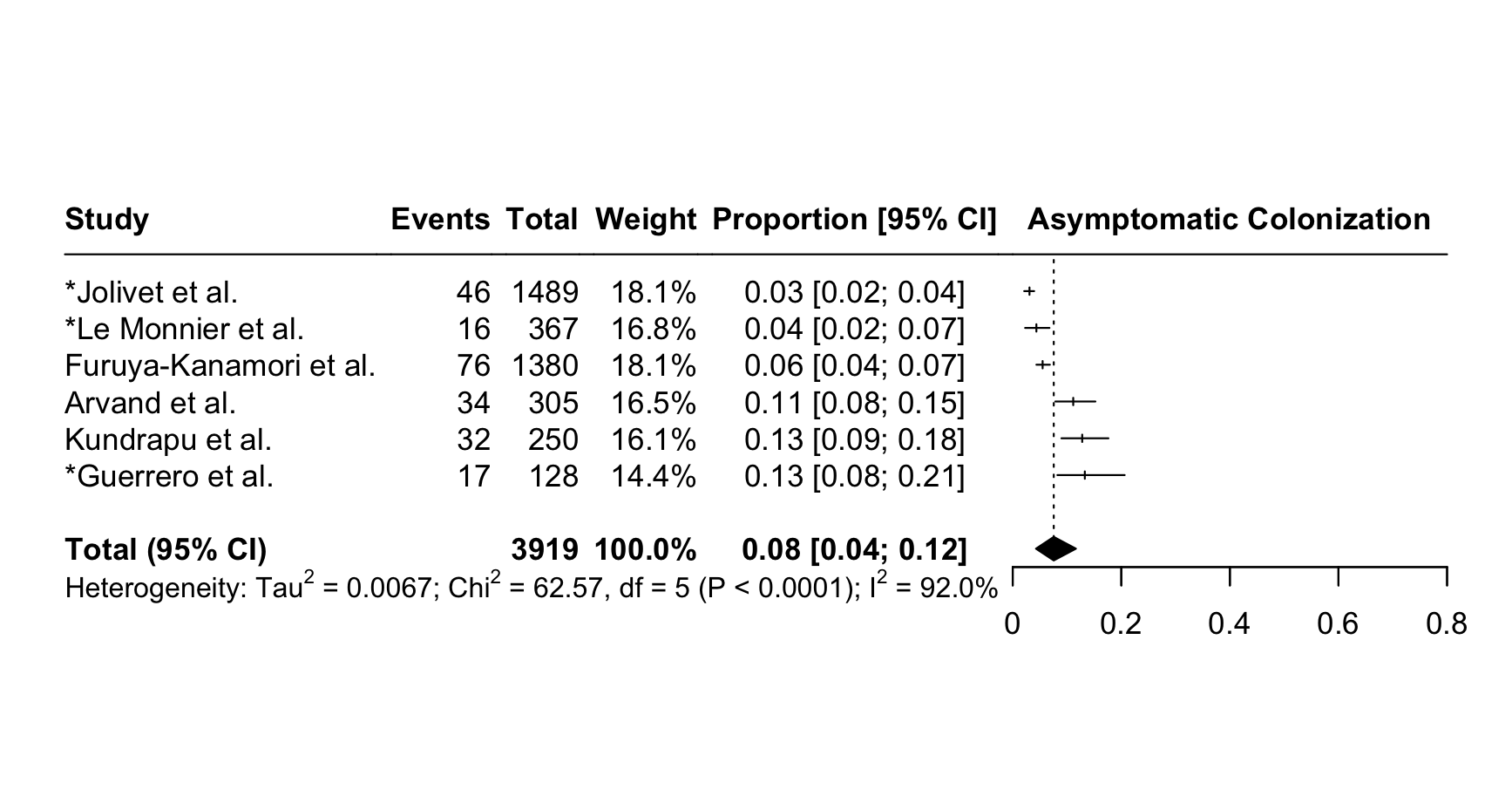


**Supplementary figure 7. Asymptomatic *C. difficile* colonization among patients at hospital admission.**

**
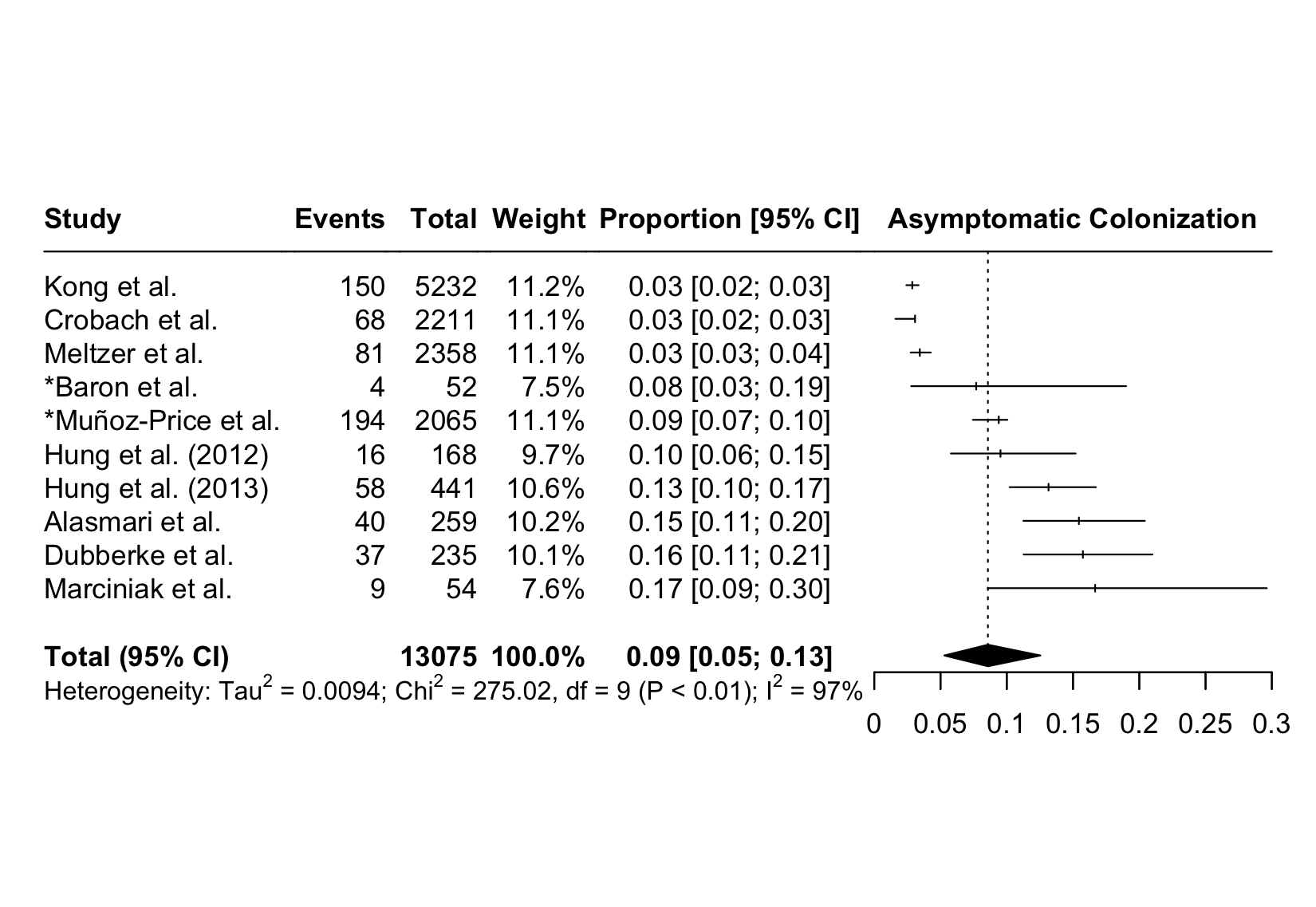
**

**Supplementary figure 8. Asymptomatic *C. difficile* colonization among patients with cystic fibrosis**


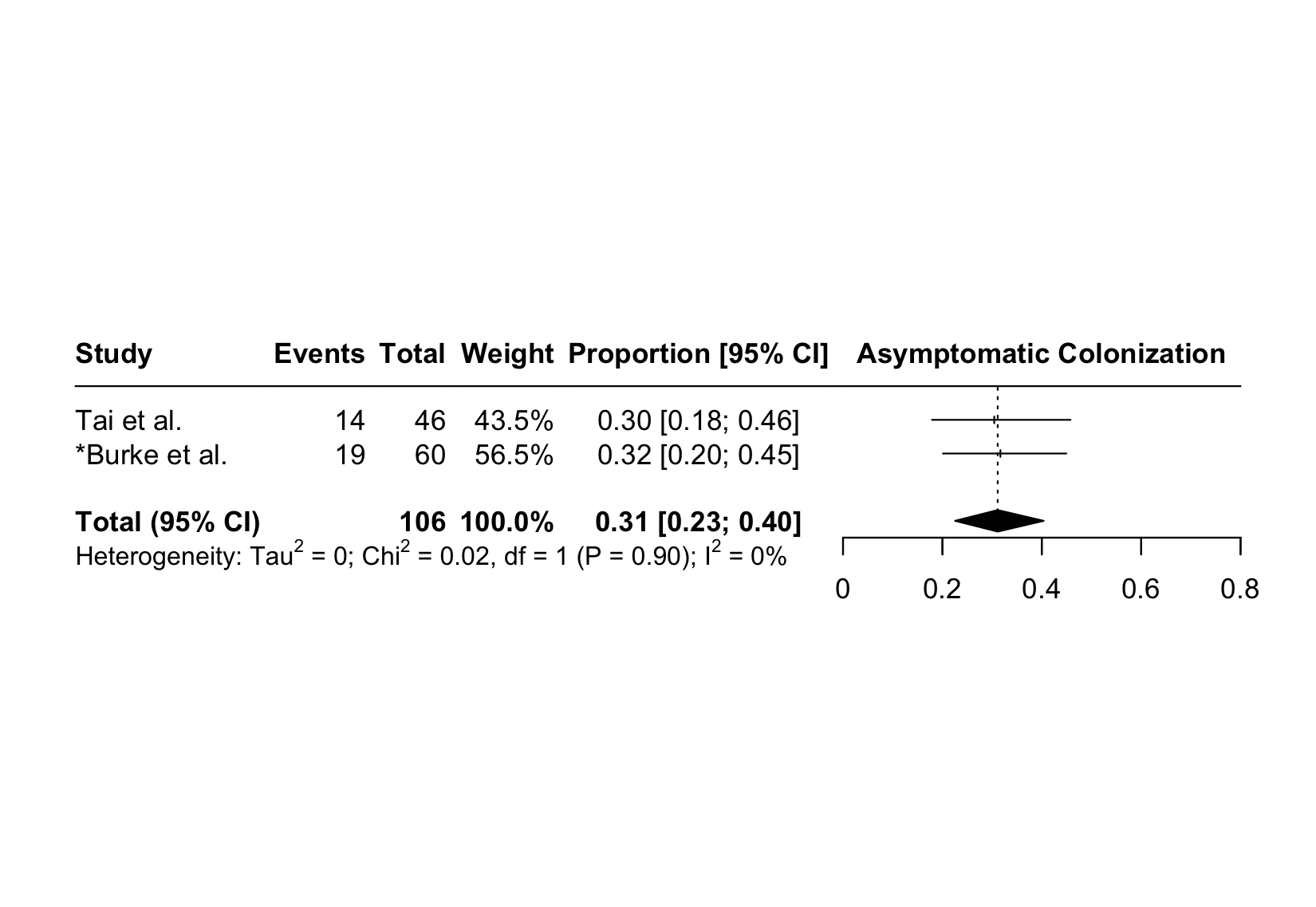


**Supplementary figure 9. Asymptomatic *C. difficile* colonization among health care workers**


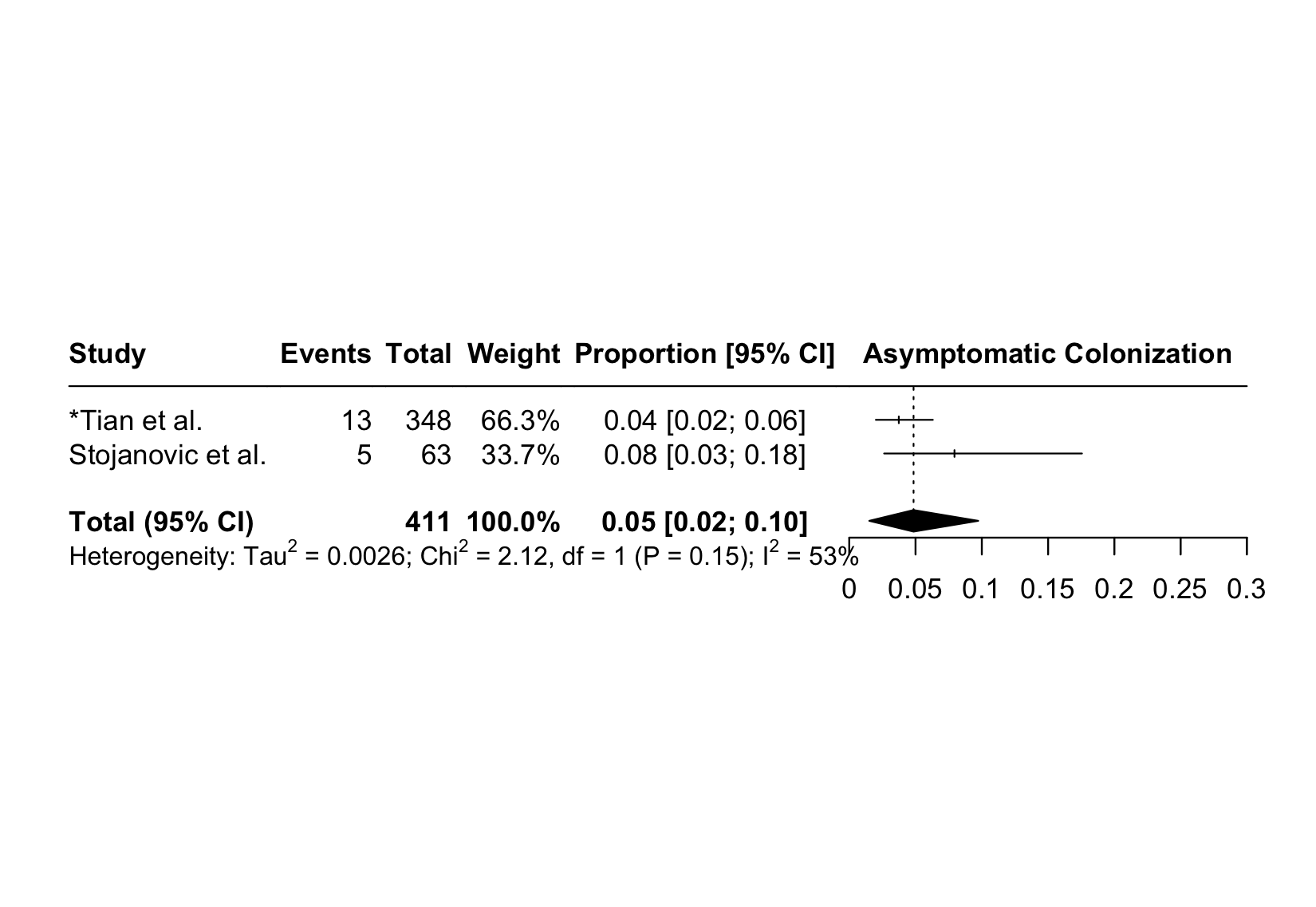


**Supplementary figure 10. Asymptomatic *C. difficile* colonization in an outbreak setting**


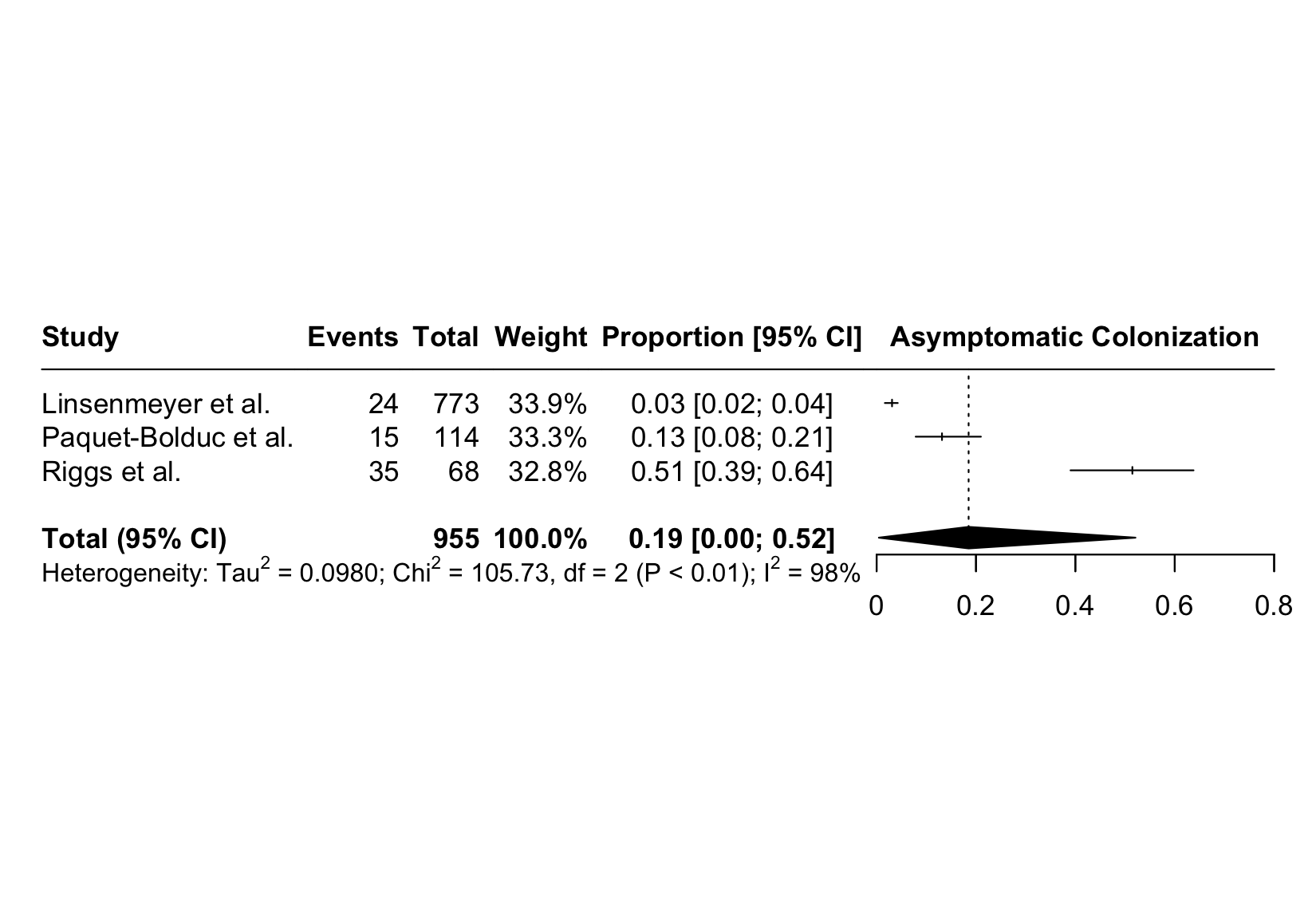

Supplement: Supplementary file 1 — Supplementary Material 1 [file 13099_2024_674_MOESM1_ESM.docx]
